# Design Optimization of a Compact Double-Ended-Tuning-Fork-Based Resonant Accelerometer for Smart Spindle Applications

**DOI:** 10.3390/mi11010042

**Published:** 2019-12-30

**Authors:** Yu-Hsuan Chen, Wei-Chang Li, Xi-Wen Xiao, Chieh-Cheng Yang, Chien-Hao Liu

**Affiliations:** 1 Department of Mechanical Engineering, National Taiwan University, Taipei 10617, Taiwan; r06522519@ntu.edu.tw (Y.-H.C.); r07522506@ntu.edu.tw (X.-W.X.); r07522530@ntu.edu.tw (C.-C.Y.); 2 Institute of Applied Mechanics, National Taiwan University, Taipei 10617, Taiwan

**Keywords:** quartz tuning fork, resonant accelerometer, double-ended tuning fork, smart spindle, chatter detection

## Abstract

With the rapid developments of the Industrial Era 4.0, numerous sensors have been employed to facilitate and monitor the quality of machining processes. Among them, accelerometers play an important role in chatter detection and suppression for reducing the tool down-time and increasing manufacturing efficiency. To date, most commonly seen accelerometers have relatively large sizes such that they can be installed only on the housing of spindles or the surfaces of workpieces that may not be able to directly capture actual vibration signals or obstruct the cutting process. To address this challenge, this research proposed a compact, wide-bandwidth resonant accelerometer that could be embedded inside high-speed spindles for real-time chatter monitoring and prediction. Composed of a double-ended tuning fork (DETF), a proof mass, and a support beam, the resonant accelerometer utilizes the resonance frequency shift of the DETF due to the bending motions of the structure during out-of-plane accelerations as the sensing mechanism. The entire structure based on commercially available quartz tuning forks (QTFs) with electrodes for symmetric-mode excitations. The advantages of this structure include low noise and wide operation bandwidth thanks to the frequency modulation scheme. A theoretical model and finite element analysis were conducted for designs and optimizations. Simulated results demonstrated that the proposed accelerometer has a size of 9.76 mm × 4.8 mm × 5.5 mm, a simulated sensitivity of 0.94 Hz/g, and a simulated working bandwidth of 3.5 kHz. The research results are expected to be beneficial for chatter detection and intelligent manufacturing.

## 1. Introduction

A great amount of effort has been invested in quantifying machine tools by employing various sensors to collect data for condition monitoring [1,2]. Among these data, vibration of machine tools may be the most useful information that can help reveal unavoidable imperfections, balance issues, and slightly bent shafts and rotors as well as provide insight into worrisome trends and even impending bearing failures [3,4]. To sense vibration in industrial machinery, accelerometers have been widely used. For example, Yao et al. [5] and Sun et al. [6] have used accelerometers to detect chatter during machining. However, these studies all rely on bulky macroscopic accelerometer modules, which often offer vibration data along only one axis and have limited installation options.

To address this issue, microelectromechanical system (MEMS) technology can be used to miniaturize the size of accelerometers and reduce the power consumption [7]. In fact, these lightweight, highly reliable, and low-cost sensors are in high demand in the era of the internet of things (IoT). The commonly used accelerometer transducer mechanisms include capacitive [8], piezoresistive [9], and piezoelectric [10,11] mechanisms, all of which produce an output voltage or charge proportional to the measured acceleration. While capacitive and piezoresistive transducers suffer from a relatively weak mechanical-electrical conversion efficiency and a higher power consumption, respectively, piezoelectric transducers surpass the other two mechanisms with a higher transducer efficiency and no standby power. For a typical piezoelectric accelerometer, acceleration will cause the proof mass to exert a force on the beam or film, creating a charge due to piezoelectric effect. However, most resonant type accelerometers have relatively large sizes and are usually installed on the housing of spindles or the stage of workpiece holders.

The goal of this research is to design a compact, resonant-type accelerometer for chatter detections. In general, the rotation speed of high-speed spindles can be as high as 30,000 rpm (i.e., 500 Hz), and chatter frequencies usually occur near the operation frequencies with several harmonics. For chatter detections, the working bandwidth of a measuring accelerometer should be 7 times the operation frequency, which is at least 3500 Hz, to cover those harmonics. In general, the resonant frequencies of accelerometers were approximately 3 times the working bandwidth (i.e., 10 kHz) [6,12]. The resonance approach has been demonstrated previously using double-ended tuning fork (DETF) structures for inertial sensors [13,14]. Analysis and finite element simulations have been carried out for some of these MEMS accelerometers [15]. Some designs are fabricated on silicon substrate and feature low temperature drift [16]. Quartz tuning forks (QTFs) are low-cost piezoelectric components often used for timing purposes, such as in quartz wristwatches. In addition, QTFs are used in various types of sensors including force sensors [17], magnetomers [18], and displacement sensors [19]. Their small size, exceptional frequency stability under temperature change, and low power consumption [20] make QTFs a great candidate for being used as resonant type accelerometers [21].

In this work, a piezoelectric DETF-based resonant accelerometer that could sense out-of-plane accelerations based on changes of the resonant frequency was proposed. In particular, the proposed resonant accelerometer consisted of a commercially available QTF, a proof mass, and a support beam, for which the QTF was attached to the entire structure forming a DETF, and the support beam was utilized to create a bending motion for the desired working bandwidth. When under an out-of-plane acceleration, the proof mass rotated due to bending of the support beam, resulting in resonance frequency shifts of the DETF. The resonant frequency shifts were exploited for sensing the out-of-plane accelerations. The advantages of using frequency modulation as the sensing scheme include high sensitivity, high stability, and requirements of simply readout circuits compared to conventional static piezoelectric accelerations. Finite element analysis was used to examine the characteristics and maximize the frequency sensitivity of the proposed DETF-based resonant accelerometer.

This paper is organized as follows. In the next section, we present the design of the DETF-based resonant accelerometer and then describe the acceleration sensing mechanism. We then present the theoretical analysis of the acceleration sensing. Subsequently, FEM analysis is conducted to examine the properties of the proposed accelerations. Important results are summarized at the end of the paper.

## 2. Device Descriptions

**Figure 1:**
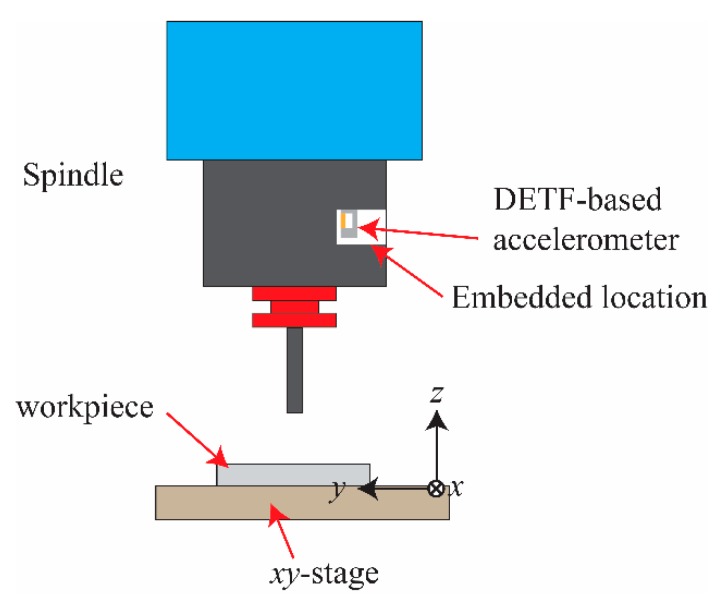
Illustration of installation of the double-ended tuning fork (DETF)-based accelerator on the smart spindle where the accelerations in the *x*- and *y*-directions could be measured for chatter detection and prediction.

In this research, we propose a DETF-based resonant accelerometer for chatter detection of high-speed spindles as shown in Figure 1. This compact accelerometer can be embedded inside the smart spindle at the same locations of the temperature probes of sensor modules [22]. The accelerometer is mounted inside the smart spindle along *z*-axis to measure the accelerations in the *x*- or *y*- directions where the coordinate system is at the moving stage of milling machines. Based on our previous work, the measured data can be exploited for chatter detections and predictions via the machine learning approach of the local outlier factor algorithm [23]. Note that in-plane indicates the plane in parallel with the QTF, which is along the *z*-direction, and out-of-plane indicates the plane normal to the QTF, which is along the *x*- or *y*-direction.

**Figure 2:**
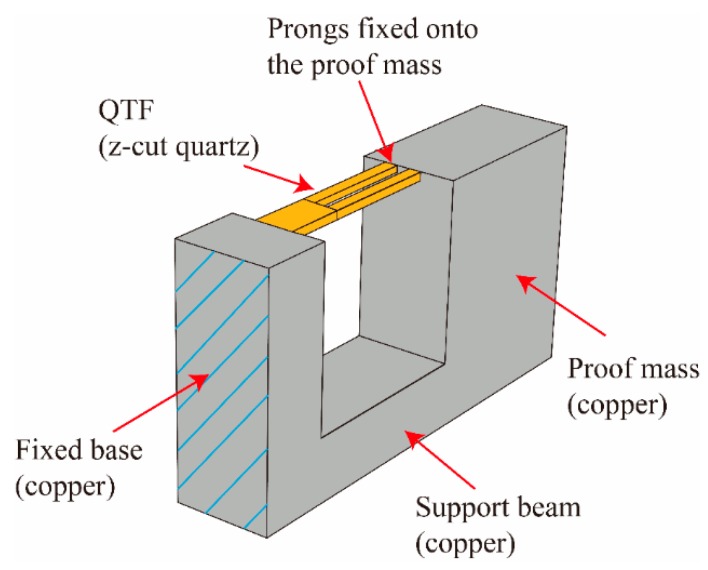
Schematic diagram of the DETF-based resonant accelerator.

The accelerometer consisted of a commercially available QTF, a proof mass, and a support beam, as shown in Figure 2. The advantage of exploiting commercially available QTF was to simplify the manufacturing process and reduce the cost. The entire structure was made of copper, and the QTF was attached to the structure to form a DETF. As mentioned before, the working bandwidth of the accelerometer for chatter detection should be larger than 3500 Hz. In contrast to conventional DETF-based accelerometers for which a proof mass is attached at the tip, a cantilever support beam was utilized in our design to create a bending motion with a much stiffer structure to extend the working bandwidth. Note that the support beam had a much higher bending stiffness than that of the DETF. When under an out-of-plane acceleration, the proof mass rotated due to bending of the lower support beam. This rotation elongated the DETF and created an axial stress in the prong, leading to a resonance frequency change for the DETF. The resonant frequency shifts were exploited for sensing the out-of-plane accelerations. In fact, the accelerometer had two resonances including the vibrations due to the applied external accelerations for creating frequency shifts of the DETF and the vibrations of the DETF driven by the external circuit for determining the accelerations. 

### 2.1. Sensing Mechanism of Acceleration

**Figure 3:**
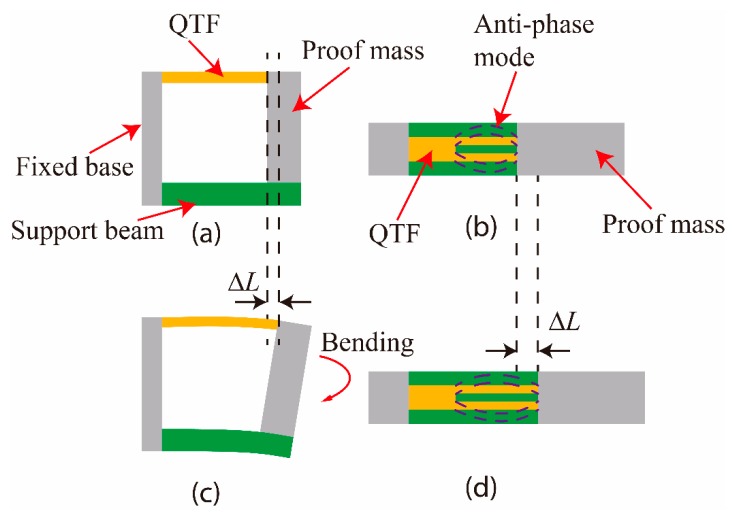
Illustration of the sensing mechanism of the resonant accelerometer. Without any acceleration, the accelerometer remained its original shape, and the DETF vibrated with the anti-phase mode driven via the external circuit: (**a**) side and (**b**) top views. During acceleration, the bending motion of the entire structure resulted in a strain of the DETF and changed the resonant frequency of the anti-phase vibrations: (**c**) side and (**d**) top views. The frequency shifts were exploited to determine the acceleration.

Figure 3 illustrates the sensing mechanism of the DETF-based resonant accelerometer. When the accelerometer was not under accelerations, it retained the original shape, and the DETF vibrated with the anti-phase mode driven via the external circuit. Figure 3a,b show the side and top views. When under an out-of-plane acceleration along the sensing axis, the proof mass rotated due to bending of the lower beam. This rotation elongated the DETF and created an axial stress in the prong, leading to a resonant frequency change of the DETF. In other words, the bending motion of the entire structure resulted in a strain in the DETF and changed the resonance frequency of the anti-phase vibrations. Figure 3c,d show the side and top views, respectively. The induced frequency shifts could be exploited to determine and sense the accelerations.

### 2.2. Quartz Tuning Fork (QTF)

**Figure 4:**
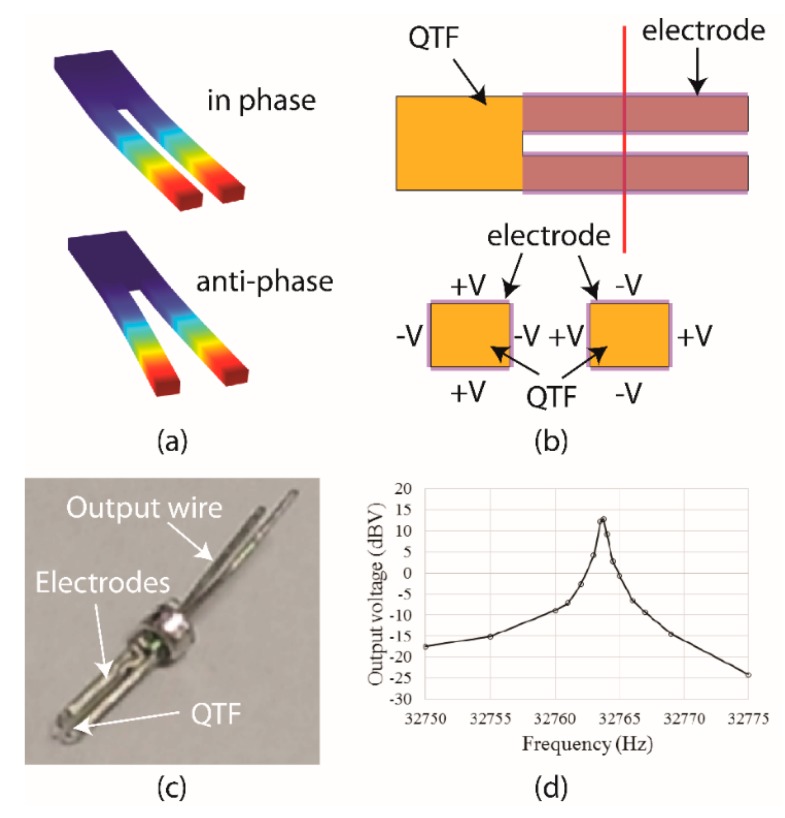
(**a**) For typical QTF resonators, there were two commonly used vibration modes including the in-phase and anti-phase modes. (**b**) To excite the anti-phase vibrations, 8 electrodes were implemented on the surfaces of the prongs of QTF excited via external driving circuits. The red line indicated the cross-section view. (**c**) The photograph of the commercial-available QTF with the resonant frequency of 32.768 kHz sealed in vacuum. (**d**) The measured frequency response of the QTF obtained in vacuum environment. For an input AC voltage of 1V, the output charge was amplified with a charge amplifier (gain = 20 GV/C), the measured Q-factor was approximately 40,000 and resonant frequency was 32.764 kHz which was lower than the spec.

QTF resonators typically have high quality factors (*Q*-factors), meaning that they resonate at precise frequencies. This makes QTFs suitable for our accelerometer design due to the requirement of precisely detecting the resonance frequency changes. In general, a QTF is composed of two quartz prongs attached to a base. Figure 4a shows two commonly seen vibration modes of QTF resonators, where the top one is the asymmetric mode (i.e., in-phase motion), and bottom one is the symmetric mode (i.e., anti-phase motion), and the two prongs oscillate in the same and opposite directions, respectively. QTF resonators are usually designed to resonate in the symmetric mode to minimize energy loss through the fixed base. External energy is required to excite the symmetric mode, and it is in the form of an external AC voltage. For each prong, there are four electrodes on its surface in the configuration shown in Figure 4b. Because quartz is a piezoelectric material, when an AC voltage is applied, an electric field is generated in the prongs. This leads to a strain in the prongs and causes vibrations.

The proposed resonant accelerometer was based on the commercially available QTF (Jin Hua Electronics Co., LTD, Taipei, Taiwan, model: 2070000000384) with Z-cut quartz and a resonant frequency of 32.768 kHz for clocking and communication systems, as shown in Figure 4c. Its frequency response was measured with an input AC voltage of 1 V, and the output charge was amplified with a charge amplifier (gain = 20 GV/C), as shown in Figure 4d. The measured *Q*-factor of the QTF sealed in vacuum was approximately 40,000 but dropped to a few thousands in air due to damping [19].

## 3. Theoey

### Frequency Deviations for Sensing Accelerations

For the proposed accelerometer, the accelerations can be obtained by measuring the frequency deviations of the DETF due to the induced axial forces under external accelerations. Studies have shown that for a single clamped-clamped beam, its natural frequency would shift when subject to external axial forces. The natural frequency of a clamped-clamped beam [24] depends on the geometric and material properties expressed as

(1) $$f=f_{0}\sqrt{1+F\times S}$$

where *F* is the axial force on the clamped-clamped beam due to the applied accelerations, and *S* depends on the geometry of the clamped-clamped beam defined as

(2) $$S=\frac{0.293L^{2}}{Etw^{3}}$$

Here, *L*, *t*, *w*, and *E* are the length, thickness, width and Young’s modulus of the clamped-clamped beam, respectively. When the resonant frequency shift, $\Delta f_{0}$, is small, it is proportional to the axial force *F*:

(3) $$\Delta f_{0}=f_{0}\sqrt{1+FS}-f_{0}\approx \frac{FSf_{0}}{2}\;\mathrm{or}\;\Delta \omega_{0}\approx \frac{FS\omega_{0}}{2}$$

Now, the analysis of the frequency deviations of a single clamped-clamped beam can be applied for analyzing DETFs. A DETF can be modeled as a spring-mass-damper system. For a DETF with anti-phase vibrations, the stiffness of each prong is larger than the coupling stiffness between two prongs. Therefore, the resonant frequency of the DETF can be obtained with the approximations of a clamped-clamped beam [24]. The equation of motion of one prong of the DETF is expressed as

(4) $$\ddot{x}+\frac{\omega_{0}}{Q}\dot{x}+\omega_{0}{}^{2}x=\frac{F_{d}(t)}{m}$$

where *x* is the displacement of one prong of the DETF, and $\omega_{0},\;Q,\;F_{d}(t),\;m$ are the natural angular frequency, quality factor, driving force, and equivalent mass of the DETF, respectively. The driving force is generated by the external voltage applied across the electrodes of the DETF. 

It is worth noting that Equation (4) applies only when the natural angular frequency $\omega_{0}$ is a constant. When the natural angular frequency varies with the time-varying axial force due to the applied accelerations, $\omega_{0}$ in Equation (4) should be replaced with ω=ω(t). Assuming that the angular frequency shift, Δω, is smaller than the natural angular frequency (i.e., $\Delta \omega \left(t\right)=\omega \left(t\right)-\omega_{0}\ll \omega_{0}$), Equation (4) is expressed as

(5) $$\ddot{x}+\frac{\omega (t)}{Q}\dot{x}+{\left[\omega_{0}+\Delta \omega (t)\right]}^{2}x\approx \ddot{x}+\frac{\omega_{0}}{Q}\dot{x}+\left[\omega_{0}{}^{2}+2\omega_{0}\Delta \omega (t)\right]x=\frac{F_{d}(t)}{m}$$

where Δω/Q and ${\left(\Delta \omega \right)}_{}^{2}$ are neglected. When the axial force is a sinusoidal function, the frequency shift must be a sinusoidal function. It can be expressed as $\Delta \omega (t)=\Delta \omega_{0}\sin(\omega_{m}t)$ for modulation angular frequency, $\omega_{m}$. Additionally, we assume that the driving force is a sinusoidal function with an angular frequency of $\omega_{0}(t)$ which is expressed as $F_{d}(t)=F_{d0}\cos(\omega_{0}t)$. Then, the above equation is the Mathieu equation:

(6) $$\ddot{x}+\frac{\omega_{0}}{Q}\dot{x}+\left[\omega_{0}{}^{2}+2\omega_{0}\Delta \omega_{0}\sin(\omega_{m}t)\right]x=\frac{F_{d0}\cos(\omega_{0}t)}{m}$$

The solution of the differential equation [24] is expressed as:

(7) $$x=x_{0}\sin(\omega_{0}t-\beta \cos(\omega_{m}t))\;\mathrm{with}\;\beta =\frac{\Delta \omega_{0}}{\omega_{m}}\;\mathrm{and}\;x_{0}=\frac{F_{d0}Q}{m\omega_{0}{}^{2}}$$

It is demonstrated that a sinusoidal axial force due to the applied acceleration can be measured by observing and demodulating the displacement signal. For practical accelerometer applications, the acceleration is, in general, a superposition of multiple sinusoidal signals. Equation (7) needs to be modified for a general input acceleration, for which the axial force can be expressed as a sum of sines at different frequencies, such as $F(t)=\sum_{i}F_{i}\sin(\omega_{i}t)$, *i* = 1, 2, 3, …, n. According to Equation (3), the frequency shift is proportional to *F*. Therefore,

(8) $$\Delta \omega (t)=\sum_{i}a_{i}\sin(\omega_{i}t)\;\mathrm{with}\;a_{i}=\frac{F_{i}S\omega_{0}}{2}$$

Since the frequency shift is assumed to be small,

(9) $${\left[\omega_{0}+\sum_{i}a_{i}\sin(\omega_{i}t)\right]}^{2}\approx \omega_{0}{}^{2}+\sum_{i}2\omega_{0}a_{i}\sin(\omega_{i}t)$$

Inserting Equation (9) into Equation (5) and repeating the steps similarly through Equation (5) to Equation (7) yields the following solution:

(10) $$x=x_{0}\sin(\omega_{0}t-\sum_{i}\beta_{i}\cos(\omega_{i}t))\;\mathrm{with}\;\beta_{i}=\frac{a_{i}}{\omega_{i}}\;\mathrm{and}\;x_{0}=\frac{F_{d0}Q}{m\omega_{0}{}^{2}}$$

**Figure 5:**
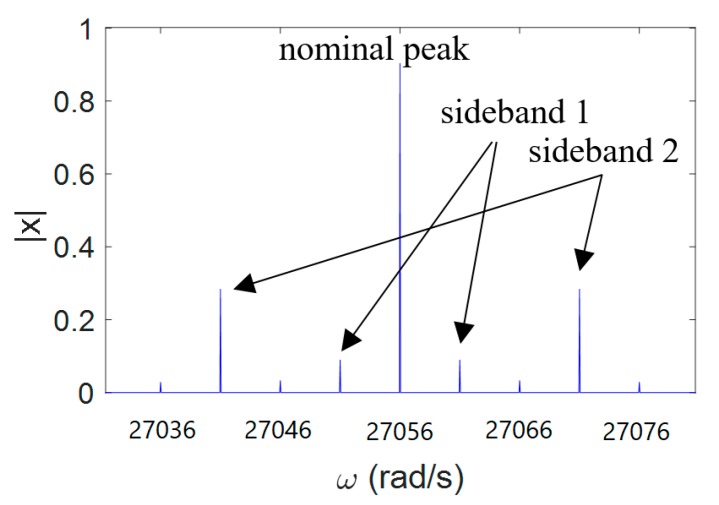
DFT of signal x=sin(27056t−0.2cos(5t)−0.6cos(15t)). A nominal peak and two pairs of sidebands can be seen at ω=27056±5 and ω=27056±15.

Notice the similarity between Equation (7) and Equation (10). In fact, Equation (7) is a special case of Equation (10) with $a_{1}=\Delta \omega_{0},\;\beta_{1}=\beta ,$ and $a_{i}=\beta_{i}=0$ for *i* > 1. When the applied acceleration is a superposition of different frequencies, the Discrete Fourier Transform (DFT) of displacement *x* exhibits multiple sidebands, each of which corresponds to its own frequency. Figure 5 illustrates this phenomenon with two modulated frequencies and two modulation indexes. The sideband magnitude and frequency are the same as those discussed in the previous section. The small peaks at the other frequencies are aliasing. Similarly, measuring the displacement could be utilized to obtain the applied accelerations with multiple frequencies. The output time-varying signal can be demodulated via frequency counting techniques [25], or the modulated parameters can be extracted via a spectrum analyzer [24].

## 4. FEM Simulations

In this research, we exploited finite element analysis to evaluate and optimize the performance of the proposed accelerometer. It was desirable to maximize the signal-to-noise ratio (SNR), meaning that under a fixed acceleration, the frequency shift of Equations (7) and (8) should be as large as possible. 

**Figure 6:**
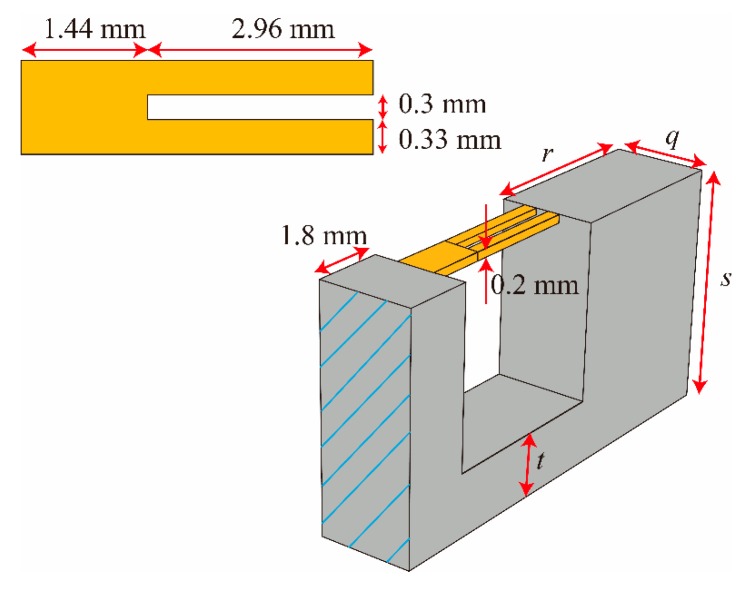
3D topology of the final design of the DETF-based resonant accelerator.

Figure 6 shows the 3D topology of the proposed accelerometer. The dimensions of the QTF used in this research were based on an off-the-shelf product for which the length, width and height of each prong were 2.96 mm, 0.33 mm and 0.2 mm, respectively, and the gap between the prongs was 0.3 mm. The base of the QTF was 1.44 mm long. For designing the accelerometer, several geometric parameters—*q*, *r*, *s* and *t*, as shown in Figure 6—were optimized with the commercial software COMSOL (V5.4, COMSOL Inc., Stockholm, Sweden). 

**Figure 7:**
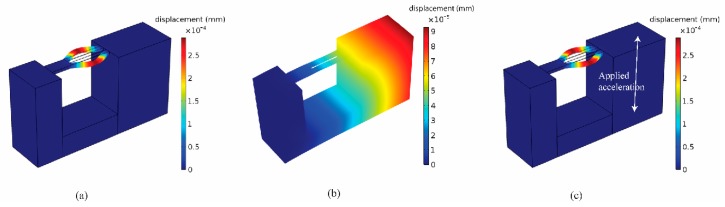
(**a**) The anti-phase resonant mode of the DETF indicating the resonance driven via the external circuit. (**b**) The fundamental resonant mode of the entire accelerometer at the first resonant frequency of 10 kHz. (**c**) The anti-phase resonant mode of the DETF under different applied accelerations where the resonant frequency of the DETF was shifted due to the applied acceleration.

**Figure 8:**
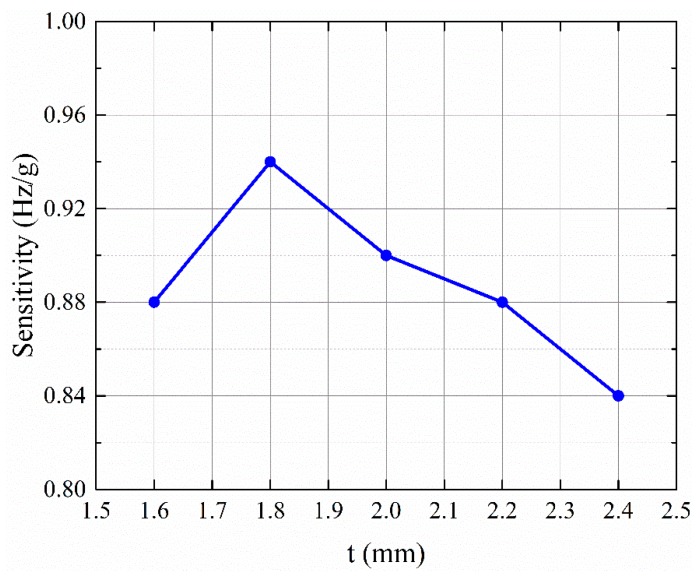
Relation between the thickness of the lower support beam and frequency shift of the DETF.

Figure 7a shows the simulated displacement of the anti-phase resonant mode of the DETF at the resonant frequency of 169.98 kHz, which verified that attaching the QTF to the structure increases the resonant frequency of the DETF from 28.9 kHz to approximately 170 kHz. Note that the anti-phase resonant mode was excited via an external driving circuit. Assuming the bending stiffness of the lower support beam was much larger than the DETF, it could be shown that *q* did not affect the frequency shifts based on Euler beam theory. One constraint of designing the accelerometer was that the first natural frequency of the entire structure should be 10 kHz, which was approximately 3 times the desired working bandwidth. Under this constraint, *s* should ideally be as large as possible, while *r* was adjusted to maintain the first natural frequency because *s* was proportional to the elongation of the QTF (and therefore proportional to the frequency shift). In practice, the desired size of the accelerometer should be as small as possible, so we chose s = 5.5 mm. There were only 2 parameters remaining to be determined: *t* and *r*. For each *t*, we chose a corresponding *r* so that the first natural frequency was 10 kHz. Based on the modal analysis, the first fundamental resonant frequency of the entire structure occurred at 10 kHz, as shown in Figure 7b, and resulted in a bandwidth of 3.5 kHz. By applying different accelerations, the frequency shift of the DETF could be obtained as shown in Figure 7c. Figure 8 shows the relation between the thickness of the lower beam and the frequency shift of the DETF. The frequency shift reached a maximum of 0.94 Hz/g when *t* = 1.8 mm, *r* = 3.56 mm, and *q* = 4.8 mm. 

For practical implementations, a thin epoxy layer of 10 μm was added between the contact surfaces of the DETF and the copper structure to study the effects of mounting DETF on copper structure with epoxy [26]. Simulation results demonstrated that the first resonant mode of the entire structure remained the same but the anti-phase resonant frequency dropped from 169.98 kHz to 157.3 kHz and the sensitivity increased from 0.94 Hz/g to 1.12 Hz/g. Although the anti-phase resonant frequency was altered due to the epoxy, it did not affect our acceleration measurement approach based on the resonant frequency shift during external accelerations.

## 5. Results

The sensitivity of the DETF-based resonant accelerometer could be increased by increasing *s* because the additional distance between the support beam and the DETF created a larger axial strain in the DETF. The drawback of increasing *s* was a larger sensor size. Another way to improve the sensitivity was to decrease the thickness of support beam *t*. However, this reduced the stiffness of the support beam and its resonant frequency, lowering the bandwidth of the accelerometer. In other words, there was a trade-off between the sensitivity, the overall size of the accelerometer, and the working bandwidth. Table 1 compares the performances of this work with that of the other resonant accelerometer. The proposed accelerometer was designed to sacrifice some sensitivity and have a wider working bandwidth for the chatter detection of smart spindles.

## 6. Conclusions

In this research, we propose a new DETF resonant accelerometers based on a commercially available quartz tuning fork (QTF) for the chatter detection of smart spindles. The analysis shows that acceleration signals can be detected in the form of frequency modulation to reduce noise and increase accuracy. Parameter optimization shows that the proposed structural designs has a good sensitivity of 0.94 Hz/g. With the improved design, which has a higher operating bandwidth compared to conventional QTF based resonant accelerometers, the results of this work show the efficacy of employing the devices in manufacturing areas to monitor the conditions of machinery and to detect and suppress chatter.

## Figures and Tables

**Table 1:** Comparison of the sensitivity with other accelerometer.

References	[27]	[28]	[29]	This Work
Sensitivity	3.4 Hz/g	18.1 Hz/g	97.86 Hz/g	0.94 Hz/g
Resonance frequency	890 kHz	348.6 kHz	149.5 kHz	170 kHz
Bandwidth	1400 Hz	566 Hz	N/A	3500 Hz
